# Molecular Characterization of the Full Kobuvirus Genome in a Cat

**DOI:** 10.1128/genomeA.00420-14

**Published:** 2014-05-01

**Authors:** Yoon-Young Cho, Seong-In Lim, Yong Kwan Kim, Jae-Young Song, Joong-Bok Lee, Dong-Jun An

**Affiliations:** a Animal and Plant Quarantine Agency, Anyang, Gyeonggi-do, Republic of Korea; b Department of Infectious Diseases, College of Veterinary Medicine, Konkuk University, Seoul, Republic of Korea

## Abstract

Kobuviruses, which belong to the family *Picornaviridae*, have been detected in fecal samples from infected animals with or without diarrhea. Here, we report the first complete genome sequence of a feline kobuvirus (FKoV) strain, FK-13, identified from the feces of a cat with diarrhea in South Korea in 2011.

## GENOME ANNOUNCEMENT

Kobuviruses (KoVs) are small nonenveloped viruses with single-stranded positive-sense genomic RNA that belong to the family *Picornaviridae*. Their genome ranges from 8.2 kb to 8.4 kb in length and has been identified in several mammalian species with or without diarrhea (1). KoVs are divided into three species: Aichi viruses, bovine KoVs, and porcine KoVs (2–4). Human Aichi viruses are the causative agents of pediatric gastroenteritis (5); however, the pathogenicity of KoVs in other mammals is unclear. Although feline kobuviruses (FKoVs) have been detected in feces from both diarrheal and normal cats (6), no complete genome sequence has been described. Here, we describe for the first time the complete genome sequence of FKoV, isolated from a stool sample collected from a South Korean cat with diarrhea in 2011.

TRIzol reagent was used to extract viral RNA from fecal suspensions (10% in phosphate-buffered saline) according to the manufacturer’s instructions. RNA was converted to cDNA using an OneStep reverse transcription (RT)-PCR kit (catalog no. 210210; Qiagen), and the 5′ and 3′ ends of the viral genome were identified by rapid amplification of cDNA ends (catalog no. K2000; Seegene). Eight sets of primers were designed based on sequences identified from canine kobuviruses (strains UK003 and USPC0082). The RT-PCR products were cloned into the pGEM-T Easy vector (Promega, USA) and then sequenced using universal primers (T7 and SP6) and an ABI Prism 3730xi DNA sequencer. All fragments were assembled and edited with Clustal X (version 1.83) to generate the final genome sequence (7). A phylogenetic tree was then constructed in MEGA 5.1 using the neighbor-joining method.

The genome of FKoV strain FK-13 is 8,201 nucleotides (nt) in length, excluding the 3′ poly(A) tail, and comprises a large open reading frame (ORF) of 7,311 nt encoding a polyprotein precursor of 2,436 amino acids, a 5′ untranslated region (UTR) of 646 nt, and a 3′ UTR of 244 nt. The genome organization is identical to that of other KoVs: a 5′ UTR, a leader protein, three structural proteins (VP0, VP1, and VP3), seven nonstructural proteins (2A to 2C and 3A to 3D), and a 3′ UTR.

A comparison with the genome sequences of several KoVs (Aichi virus, porcine KoV, canine KoV, and bovine KoV) deposited in the GenBank database revealed that FK13 has 75.3% nucleotide sequence homology with Aichi virus strain A846/88 (accession no. AB040749 and NC001918), 80.6% homology with canine KoV strain UK003 (accession no. KC161964), 63.1% homology with porcine KoV strain swine/S-1-HUN/2007/Hungary (accession no. EU787450 and NC011829), and 62.2% homology with bovine KoV strain U-1 (accession no. AB084788 and NC004421).

A phylogenetic tree based on ORF sequences derived from KoVs isolated from several mammalian species indicated that FKoV strain FK-13 is most closely related to canine KoVs and mouse KoVs.

To the best of our knowledge, this is the first report of the complete genome sequence of FKoV. The FKoV strain FK-13 sequence will help us to better understand the genetic diversity of kobuviruses and facilitate further study of the epidemiology and pathogenicity of FKoVs.

### Nucleotide sequence accession number.

The complete genome sequence of FKoV strain FK-13 was deposited in GenBank under accession no. KF831027.
